# An Unusual Cause of Bleeding in a Patient with Chronic Myeloid Leukemia Chronic Phase

**DOI:** 10.1155/2019/5674193

**Published:** 2019-09-09

**Authors:** S. Kartthik, Prakas K. Mandal, Saleh Mohammed Abdullah

**Affiliations:** ^1^Department of Haematology–Oncology, GKNM Hospital, Coimbatore, Tamilnadu, India; ^2^Department of Haematology, NRS Medical College and Hospital, Kolkata, West Bengal, India; ^3^Department of Medical Laboratory, Faculty of Applied Medical Sciences, Jazan University, Jazan, Saudi Arabia

## Abstract

Chronic myelogenous leukemia (CML) is a clonal myeloproliferative neoplasm (MPN) characterized by dysregulated and uncontrolled proliferation of mature and maturing granulocytes with normal differentiation. A genetic hallmark of CML is the presence of the fusion gene product BCR-ABL. Bleeding diathesis in CML patients is rare (<10%) and primarily caused by acquired platelet dysfunction. We report a rare case of an adult CML chronic phase patient who presented with spontaneous muscle hematoma due to acquired Glanzmann's thrombasthenia (GT). On laboratory workup, a GT was confirmed along with the diagnosis of CML in chronic phase. The muscle hematoma was completely resolved following imatinib therapy. The present case demonstrates that bleeding is a complication of MPNs and highlights the importance of both acquired GT diagnosis to determine the cause of bleeding in CML and of prompt treatment with imatinib to reverse this condition.

## 1. Introduction

Chronic myeloid leukemia (CML) is a clonal hematopoietic stem cell disorder driven by the BCR-ABL1 chimeric gene product and resulting from a reciprocal balanced translocation between the long arms of chromosomes 9 and 22, t(9;22), cytogenetically detected as the Philadelphia chromosome (Ph). Hemorrhagic manifestations are not uncommon in CML patients. Platelet dysfunction appears to be the primary cause of bleeding in patients with CML. Hemorrhagic manifestations typically resolve with treatment, suggesting that the platelet dysfunction is related to disease activity [1]. Acquired Glanzmann's thrombasthenia (GT) is a rare bleeding disorder characterized by abrupt onset of moderate-to-severe bleeding tendency and prolonged bleeding time but with normal platelet count and normal or reduced platelet glycoprotein (GP) expression. Acquired GT is an uncommon event in association with CML, and it may be caused by the formation of autoantibodies against the GPIIb/IIIa complex [2]. Nurden has reported that platelet glycoprotein dysfunction and signaling defects may occur in myeloproliferative neoplasms (MPNs), including CML [3].

We report a rare case of adult CML chronic phase in a patient who presented with spontaneous muscle hematoma due to acquired GT; however, due to constraints, glycoprotein expression was not measured. The present case demonstrates that bleeding is a complication of MPNs and emphasizes the importance of acquired GT diagnosis to identify the cause of bleeding in CML; furthermore, prompt treatment with imatinib can achieve a reversal of this condition.

## 2. Case Presentation

A 45-year-old married female patient presented with swelling in the right thigh and low-grade intermittent fever for the past month with no history of trauma. The patient had a history of two small hematomas in the right and left thigh region in the preceding 3 months that had resolved spontaneously. There was no family history of bleeding disorder, and she was not taking any prior medication. On examination, she had normal vital status with mild pallor, mild hepatomegaly, and moderate splenomegaly. Ultrasonography of the abdomen showed hepatomegaly with a liver span of 16.1 cm and massive splenomegaly of 14 cm below the left costal margin, while ultrasonography of the right thigh showed extensive hematoma on the anterolateral aspect limited in the upper-mid region of both intramuscular and fascial planes.

Complete hemogram with peripheral blood smear showed Hb 8.5 gm/dl, hematocrit 20.0%, MCV 82.3 fl, MCH 35.0 pg, MCHC 42.5 gm/dl, platelet count 303 × 10^3^/*μ*l, and a very high leukocyte count of 193 × 10^9^/l with neutrophil 41%, lymphocyte 1%, basophil 2%, myelocyte 37%, metamyelocyte 14%, blast cell 5%, 4 nRBC/100 WBC, and reticulocyte count 4.9%. Bone marrow aspiration (BMA) revealed hypercellular marrow spaces with increased granulocytic precursors, basophils, and eosinophils. Chromosomal analysis of the BMA specimen showed 46 XX t(9; 22) (q34;q11.2) in 100% of the 20 metaphase cells examined. Reverse transcription real-time multiplex PCR was positive for the BCR-ABL1 fusion transcript p210. Based on the peripheral blood film, BMA findings, and molecular studies, a diagnosis of CML chronic phase was established.

**Figure 1:**
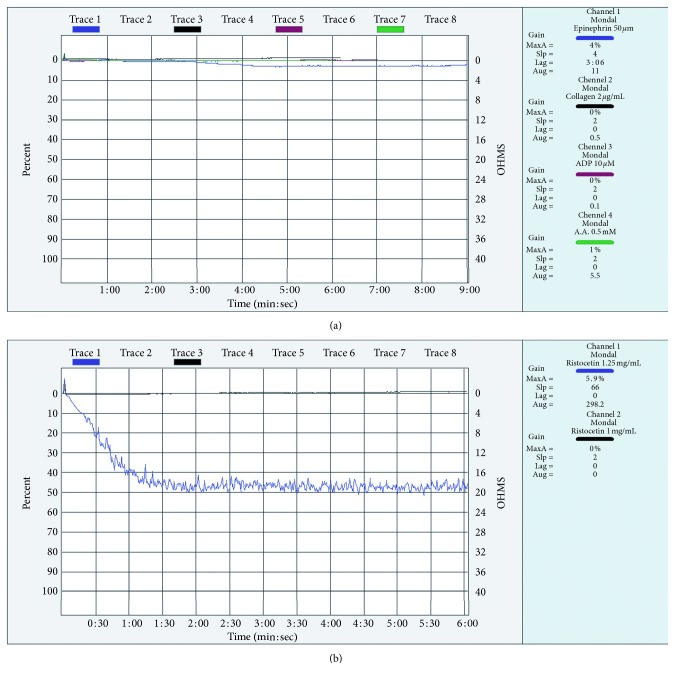
Platelet aggregation test by LTA at the time diagnosis. (a) Platelet aggregation test with collagen, ADP, thrombin, and arachidonic acid shows a complete absence of platelet aggregation. (b) Platelet aggregation test with ristocetin shows a normal response.

Considering a bleeding diathesis in a CML patient, we then attempted to identify the etiology. The screening coagulation tests included bleeding time (BT) 14 min; prothrombin time (PT) 13 sec; activated partial thromboplastin time (aPTT) 29 sec, and fibrinogen level 260 mg/dl. She had normal PT, aPTT, and fibrinogen levels. The clot was insoluble at 37°C in 24 hr incubation, but her bleeding time was prolonged. A von Willebrand factor (vWF) assay was performed and showed a value >120%. Platelet function testing revealed no platelet aggregation with ADP (10 *μ*M), epinephrine (50 *μ*M), collagen (2 *μ*g/mL), arachidonic acid (0.5 mM), and normal ristocetin aggregation (1.25 mg/mL and 0.5 mg/mL; Figure 1). The pattern of platelet aggregation response was suggestive of an acquired GT, such as a defect in a case of CML chronic phase. However, an ELISA (AssayMax™ Human GPIIb/IIIa ELISA Kit, Assaypro, St. Charles, MO) for anti-GPIIb/IIIa antibody titer was negative.

**Figure 2:**
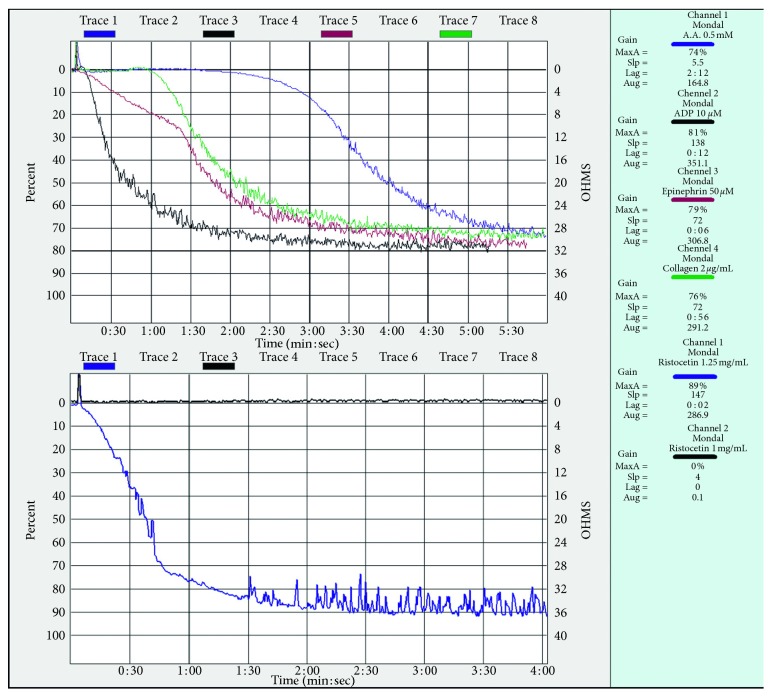
Platelet aggregation study by LTA at 3 months follow-up showing a normal response to all of the agonists.

The patient was started on imatinib mesylate 400 mg once daily after obtaining the BCR-ABL report. Bleeding was managed initially with tranexamic acid and intranasal desmopressin. Detection of a GT-like condition did not change the course of management in this patient. Clinical reversal of bleeding was noted following imatinib therapy. After 1 month of therapy, the swelling completely disappeared. Her platelet function test, repeated after 3 months, showed normalization of response to all of the agonists (Figure 2). The patient attained major molecular response (MMR) at 3 months. This patient did not show any bleeding manifestation until the most recent follow-up (i.e., 18 months after MMR).

## 3. Discussion

A qualitative or quantitative defect—either congenital or acquired—of either GPIIb or GPIIIa is the essential biochemical basis for GT. GPIIb/IIIa is a calcium-dependent heterodimer that binds preferentially to fibrinogen or vWF, but it also binds to fibronectin or vitronectin. Consequently, GT is characterized by defective *in vitro* platelet aggregation and a lifelong bleeding tendency. Platelet dysfunction, whether in the form of platelet hypofunction or hyperfunction, in chronic myeloproliferative disorder is multifactorial in cause. Specific platelet defects, including abnormal platelet morphology, acquired storage pool disease, platelet membrane abnormalities, and abnormal arachidonic acid metabolism, have previously been described [4].

Several mechanisms of projecting GT phenotype in CML, such as defects in a signaling pathway vital for *α*IIb*β*3 activation [5], defects in activation-dependent inside-out signaling [6], dysfunction in the phosphatidylinositol-3 kinase (PI3 kinase)/Rap1/*α*IIb*β*3 pathway [7], poor platelet aggregation attributed to dysregulated tyrosine kinase activity associated with BCR-ABL [8], and reduced *α*IIb*β*3 on platelets [9] have been described. In CML, the platelet dysfunction is believed to have originated from a clonal expansion of dysfunctional megakaryocytes. Thus, the treatment targeting BCR-ABL would be equally effective in reducing the CML blasts and dysfunctional megakaryocytes. This is corroborated by the observation that employing tyrosine kinase inhibitors for use in patients with CML could improve platelet dysfunction [5]. There exist very few case reports of soft tissue hematoma, such as spontaneous mediastinal hematoma, hematoma in iliac psoas muscle, spinal epidural hematoma, and acute subdural hematoma, as the initial presenting features of CML [10–12].

An extensive review of the literature revealed that very few cases of acquired GT have been reported in association with neoplasms of lymphoid origin such as multiple myeloma, non-Hodgkin's lymphoma, and Hodgkin's disease [13]. Kannan et al. found a case of hairy cell leukemia associated with acquired GT [14]. Although bleeding diathesis attributed to defective platelet function is not uncommon in CML, typical GT (e.g., a defect in platelet aggregation) has not been reported earlier in adult CML. A singular case was reported in the literature by Chauhan et al. of pediatric CML associated with a GT-like defect [9] due to dysfunctional circulating platelets.

The present case highlights the importance of thorough investigation in a CML patient who presents with muscle hematoma and of using appropriate coagulation studies since acquired GT as a cause often remains underdiagnosed. Caution is warranted because imatinib itself has been reported to have a negative effect on platelet aggregation; it has been associated with bleeding diathesis. Hence, regular follow-up is needed after imatinib therapy to examine any clinical evidence of bleeding and monitor platelet function. This patient is on regular follow-up and did not manifest any bleeding until this visit.

In conclusion, our report demonstrates that soft tissue hematoma may be a presenting feature of CML due to platelet dysfunction. Poor platelet aggregation in this case is possibly attributed to dysregulated tyrosine kinase activity associated with BCR-ABL; treatment with imatinib, which targets this kinase, restored the aggregation response. Bleeding manifestation can occur in <10% of CML patients with or without imatinib therapy. The concordance of hematoma formation with signs and symptoms of MPN suggests bleeding secondary to MPN. Moreover, basic coagulation profile, platelet aggregation studies, and autoantibodies to platelet glycoprotein are recommended to identify the cause of bleeding in CML patients [9]. Successful treatment of underlying CML reduces the risk of life-threatening bleeding diathesis. Advanced studies and research would further help to identify platelet, blood cell, plasma, and vascular gene variants that may influence bleeding in MPN.
