# Corrigendum to “An Unusual Cause of Bleeding in a Patient with Chronic Myeloid Leukemia Chronic Phase”

**DOI:** 10.1155/2020/2194247

**Published:** 2020-08-30

**Authors:** S. Kartthik, Prakas K. Mandal, Souren Pal, Saleh Mohammed Abdullah

**Affiliations:** ^1^Department of Haematology-Oncology, GKNM Hospital, Coimbatore, Tamilnadu, India; ^2^Department of Haematology, NRS Medical College and Hospital, Kolkata, West Bengal, India; ^3^Department of Medicine, NRS Medical College and Hospital, Kolkata, West Bengal, India; ^4^Department of Medical Laboratory, Faculty of Applied Medical Sciences, Jazan University, Jazan, Saudi Arabia

In the article titled “An Unusual Cause of Bleeding in a Patient with Chronic Myeloid Leukemia Chronic Phase” [1], the author Dr. Souren Pal has been added to the authors list as he had involved in the initial patient care, i.e., clinical study partly. The final author list is S. Kartthik, Prakas K. Mandal, Souren Pal, and Saleh Mohammed Abdullah.
